# Validation and Invalidation of Chemical Probes for the Human *N*-myristoyltransferases

**DOI:** 10.1016/j.chembiol.2019.03.006

**Published:** 2019-06-20

**Authors:** Wouter W. Kallemeijn, Gregor A. Lueg, Monica Faronato, Kate Hadavizadeh, Andrea Goya Grocin, Ok-Ryul Song, Michael Howell, Dinis P. Calado, Edward W. Tate

**Affiliations:** 1 Department of Chemistry, Imperial College London, Molecular Research Science Hub, 80 Wood Lane, London W12 0BZ, UK; 2 The Francis Crick Institute, 1 Midland Road, London NW1 1AT, UK; 3 Peter Gorer Department of Immunobiology, School of Immunology & Microbial Sciences, King's College London, London SE1 9RT, UK

**Keywords:** *N*-myristoylation, *N*-myristoyltransferases (NMT), 2-hydroxymyristic acid, D-NMAPPD (B13), Tris-DBA palladium, IMP-366 (DDD85646), IMP-1088, metabolic tagging, sortase A ligation, chemical proteomics

## Abstract

On-target, cell-active chemical probes are of fundamental importance in chemical and cell biology, whereas poorly characterized probes often lead to invalid conclusions. Human *N*-myristoyltransferase (NMT) has attracted increasing interest as target in cancer and infectious diseases. Here we report an in-depth comparison of five compounds widely applied as human NMT inhibitors, using a combination of quantitative whole-proteome *N*-myristoylation profiling, biochemical enzyme assays, cytotoxicity, in-cell protein synthesis, and cell-cycle assays. We find that *N*-myristoylation is unaffected by 2-hydroxymyristic acid (100 μM), D-NMAPPD (30 μM), or Tris-DBA palladium (10 μM), with the latter compounds causing cytotoxicity through mechanisms unrelated to NMT. In contrast, drug-like inhibitors IMP-366 (DDD85646) and IMP-1088 delivered complete and specific inhibition of *N*-myristoylation in a range of cell lines at 1 μM and 100 nM, respectively. This study enables the selection of appropriate on-target probes for future studies and suggests the need for reassessment of previous studies that used off-target compounds.

## Highlights

• IMP-1088 selectively blocks *N*-myristoylation in cells at low nanomolar concentration
• Three widely used human NMT probes are invalidated by 6 orthogonal assay modalities
• 2-Hydroxymyristate is inactive up to 100 μM; D-NMAPPD is non-specifically cytotoxic
• Tris-DBA palladium does not inhibit NMT in cells, and is cytotoxic by precipitation

## Graphical Abstract

**Figure:**
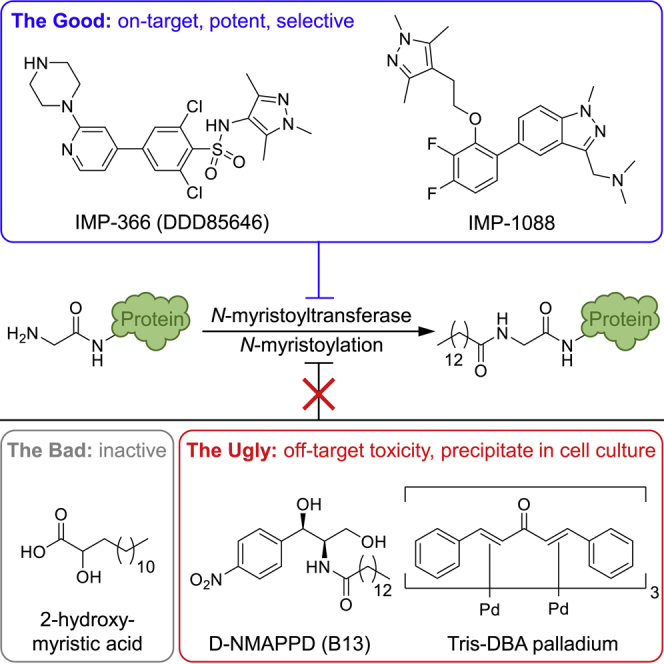

## Teaser

Specific inhibitors of protein *N*-myristoylation are powerful probes for exploring the role of this post-translational modification in cells and have therapeutic potential. Kallemeijn et al. identify three flawed but commonly applied human *N*-myristoyltransferase inhibitors, and advocate two classes of high-affinity inhibitors as probes with a well-validated on-target cellular mode of action.

## Introduction

**Figure 1:**
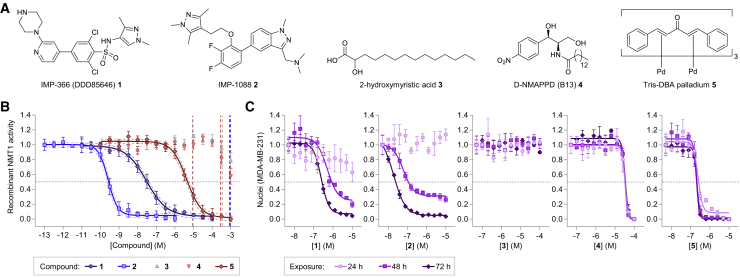
Effects on Recombinant Human NMT1 and Cytotoxicity in Living Cells (A) Molecular structures of IMP-366 **1**, IMP-1088 **2**, 2-hydroxymyristic acid **3**, D-NMAPPD **4**, and Tris-DBA palladium **5**. (B) Inhibition of recombinant human NMT1 by **1–5**. Mean of n = 2 experiments performed in triplicate, ±SEM. Four-parametric variable slope function fitted with the 95% confidence interval is shown (continuous and dotted lines, respectively). Vertical dotted lines indicate presence of precipitates. (C) Nuclei counts of MDA-MB-231 cells after exposure to **1**–**5** for 24, 48, and 72 h. Mean of n = 4 measurements ± SEM.

*N*-Myristoylation is the irreversible covalent attachment of a saturated 14-carbon myristic acid to the N-terminal glycine of more than 200 proteins in the human proteome (Wright et al., 2010, Lanyon-Hogg et al., 2017, Castrec et al., 2018). The majority of *N*-myristoylation occurs co-translationally at the ribosome, where removal of initiator methionine exposes an N-terminal glycine and subsequent recognition sequence (Johnson et al., 1994, Castrec et al., 2018), while post-translational *N*-myristoylation may occur following endoproteolysis, for example by caspases during apoptosis, to reveal a *de novo* N-terminal glycine amenable to modification (Utsumi et al., 2003, Thinon et al., 2014). In humans, *N*-myristoylation is catalyzed by two myristoyl-coenzyme A (CoA):protein *N*-myristoyltransferases (NMTs), NMT1 and NMT2 (Dyda et al., 2000; Giang and Cravatt, 1998, Castrec et al., 2018), which share ∼77% sequence identity and have overlapping biological functions and protein substrate selectivity (Giang and Cravatt, 1998, Castrec et al., 2018). NMT knockout studies in mice have illustrated the importance of *N*-myristoylation for T cell development and activation (Rampoldi et al., 2012), and NMT inhibition has been shown to induce ER stress, cell-cycle arrest, and apoptosis in cancer cell lines (Thinon et al., 2016). *N*-Myristoylated proteins play a pivotal role in pathological processes underlying infections by viruses and protozoa (Lanyon-Hogg et al., 2017), with multiple studies validating NMTs as a pharmacological target for the development of anti-viral (Mousnier et al., 2018, Corbic Ramljak et al., 2018), anti-parasitic (Price et al., 2003, Frearson et al., 2010, Herrera et al., 2016, Wright et al., 2014), and anti-fungal drugs (Tate et al., 2014, Weinberg et al., 1995). A limited set of compounds have been reported as inhibitors of human NMTs, with the most commonly applied being IMP-366 (DDD85646) **1**, IMP-1088 **2**, 2-hydroxymyristic acid **3**, D-NMAPPD **4**, and Tris-DBA palladium **5** (Figure 1A). To date there have been no reports of NMT1/NMT2-selective inhibitors, likely due to the very high sequence conservation between their catalytic domains. IMP-366 **1** (DDD85646) is a nanomolar inhibitor of both recombinant (∼20 nM half-maximal inhibitor concentrations [IC_50_]) and cellular human NMTs (Frearson et al., 2010). We recently reported that IMP-1088 **2**, an improved inhibitor (∼200 pM *K*_D_) of both NMTs (Mousnier et al., 2018; Figure 1A). At low nanomolar concentrations, **2** completely inhibited *N*-myristoylation in cells, including that of a rhinoviral capsid protein, thus preventing infectious virus formation (Mousnier et al., 2018). High-resolution X-ray crystal structures further support the high affinity and selectivity of **1** and **2** for human NMT1 and NMT2 (Thinon et al., 2014, Mousnier et al., 2018). Myristic acid analog 2-hydroxymyristic acid **3** (Figure 1A) was the first compound reported to inhibit NMT in cellular studies (Paige et al., 1989, Paige et al., 1990). At 100 μM to 1 mM, **3** has been reported to reduce metabolic labeling of cells with [^3^H]myristate (Vaandrager et al., 1996, Tan et al., 2016) and has been suggested to prevent NMT-dependent viral budding (Perez et al., 2004, Du et al., 2010, Tan et al., 2016). D-NMAPPD **4**, also known as B13 (Figure 1A), was first reported as an inhibitor for lysosomal acid ceramidase, inducing ceramide-driven apoptosis in melanoma (Bielawska et al., 1996, Raisova et al., 2002). Recent work, however, has claimed that **4** also inhibits NMT in cells (Kim et al., 2017), with inhibition hypothesized to depend on the 14-carbon chain shared between **4** and myristoyl-CoA, and by various putative interactions proposed through *in silico* docking. **4** has a reported biochemical human NMT1 IC_50_ of 78 μM which, surprisingly, is ca. 5-fold higher than its reported capacity to reduce *N*-myristoylation in living cells (IC_50_ ∼15 μM) (Kim et al., 2017). Tris-DBA palladium **5** (Figure 1A) has been described as an NMT inhibitor (Bhandarkar et al., 2008), although it is more generally used as a catalyst in Suzuki cross-coupling reactions in organic chemistry (Kudo et al., 2006). **5** has been reported to have anti-tumor activity against A375 melanoma, as exposure of cells to 11 μM **5** resulted in a 96% decrease in cell count (Bhandarkar et al., 2008). In more recent work, **5** has been shown to cause apoptosis in multiple myeloma (de la Puente et al., 2016), and to kill chronic lymphocytic leukemia B cells (Kay et al., 2016) and pancreatic cancer cells (Díaz et al., 2016).

The importance of well-characterized, on-target cell-active chemical probes for proteins is widely appreciated across the fields of chemical and cell biology (Arrowsmith et al., 2015, Blagg and Workman, 2017). Here we report a comprehensive and critical evaluation of the five compounds most prominently claimed and applied as human NMT inhibitors to determine whether they are fit for purpose as chemical probes. We find that compounds **3**, **4**, and **5** act off-target in all cell lines tested and provide robust evidence that they do not inhibit *N*-myristoylation in a concentration range consistent with activity on NMT; these data directly contradict prior studies, which have therefore incorrectly applied these compounds as NMT inhibitors. Compounds **1** and **2** were confirmed as the only agents in this panel with potent on-target NMT inhibitory activity in cells, making them the current best-in-class tools for future studies of human *N*-myristoylation.

## Results

### Effects on Recombinant Human NMT1 and Cell Line Cytotoxicity

We first examined recombinant human *N*-myristoyltransferase 1 (rNMT1) inhibition by compounds **1**–**5**, using a standard assay that measures generation of CoA thiol by-product (Goncalves et al., 2012) (Figure 1B). rNMT1 was inhibited in a concentration-dependent manner by **1** and **2**, with IC_50_ of 29 and 0.24 nM respectively; these values are consistent with previously published data (Table 1, Goncalves et al., 2012, Mousnier et al., 2018), although rNMT1 is present at 5 nM in the assay and therefore the IC_50_ for **2** cannot be precisely quantified. rNMT1 activity was not reduced by **3** or **4** below 300 μM (Figure 1B), with extrapolated IC_50_ values higher than those previously reported (Table 1). Tris-DBA palladium **5** reduced rNMT1 activity in a concentration-dependent manner, with an IC_50_ of 4.2 μM. We noted that dilutions of **5** suffered from insolubility in aqueous solutions, as illustrated by black aggregates (Figure S1A); absorbance at 750 nm (a measure of degree of precipitation) was determined at various concentrations of **1**–**5** (Figure S1B), revealing that **5** precipitated at 10 μM in large (>750 nm) particles, followed by **3** and **4** at 300 μM. The correlation between reduction of rNMT1 activity and precipitation suggests that NMT inhibition by **3–5** may be partially caused by non-specific interactions with the enzyme. Precipitation of **1** and **2** occurred above 300 μM, 4–6 orders of magnitude above their respective IC_50_ values (Figure 1B).

**Table 1:** Determined *In Vitro* IC_50_ Values for Compounds **1–5** Compared with Reported Values

Compound	*In Vitro* IC_50_	
	Reported	This Article
IMP-366 **1**	3–22 nM^a^	29 nM
IMP-1088 **2**	<1 nM^b^	0.35 nM
2-hydroxymyristic acid **3**	100 μM^c^	>300 μM
D-NMAPPD **4**	77 μM^d^	>300 μM
Tris-DBA palladium **5**	0.5–1.3 μM^e^	4.2 μM

a Frearson et al., 2010.

b Mousnier et al., 2018.

c Paige et al., 1990.

d Kim et al., 2017.

e Bhandarkar et al., 2008.

Next, we determined cytotoxicity for **1**–**5** in cell lines of breast (MDA-MB-231) and cervical (HeLa) cancer. Cells were exposed for 24, 48, or 72 h to a range of concentrations in **1–5**, followed by nuclei counting as a measure for proliferation, while metabolic activity was assessed using a fluorescent Cell Titer Blue assay using NAD(P)H-dependent cellular oxidoreductase activity to generate an insoluble, fluorescent resorufin product (Stepanenko and Dmitrenko, 2015). **1** and **2** affected proliferation of MDA-MB-231 cells only after 48 and 72 h and in a concentration-dependent manner (Figure 1C), in line with previously published observations (Thinon et al., 2016, Mousnier et al., 2018). Proliferation was unaffected by **3**, while 30 μM **4** hindered proliferation equally at all time points. In a time- and concentration-dependent manner, **5** potently decreased proliferation with a ∼300 nM IC_50_. Metabolic activity of MDA-MB-231 cells was affected in line with viability by **1–5** (Figure S1C). HeLa cells proved less sensitive to **1**–**5**, but overall trends were analogous to those in MDA-MB-231, both for proliferation and metabolic activity (Figures S1D and S1E, respectively). Tris-DBA palladium **5** precipitated in the media, forming black rod-shaped crystals that adhere to the growth substrate (Figure 4F). To investigate whether the crystals were the cytotoxic component, we analyzed the cytotoxicity of **1–5** to Jurkat cells, a T cell leukemia cell line that grows in suspension. Jurkat cells responded to **1**, **2**, **3**, and **4** in a manner highly similar to that in for MDA-MB-231 and HeLa (Figure S1F). In sharp contrast, **5** had no effect on metabolic activity in Jurkat, even at the most cytotoxic conditions for MDA-MB-231 and HeLa cells (10 μM **5** for 72 h, Figures 1C and S1E, respectively). The absence of cytotoxicity strongly suggests that **5** kills cells through proximal interactions between adhering cells and crystals.

### Effects on *N*-Myristoylation, NMT Expression, and NMT Substrate ARL1 in Living Cells

**Figure 2:**
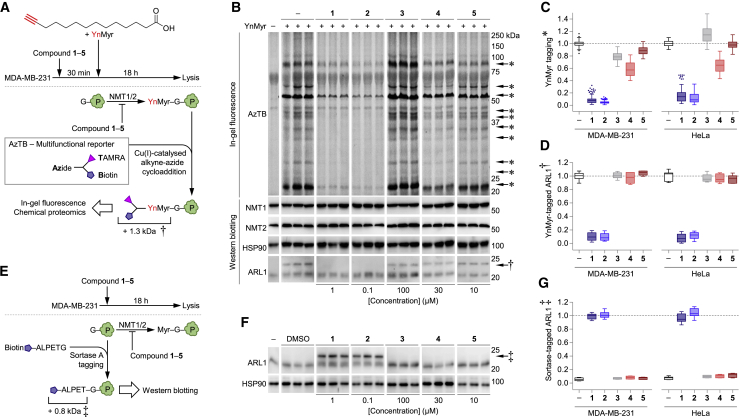
Effects on Cellular *N*-myristoylation, *N*-myristoyltransferases, and Substrate ARL1 in Living Cells (A) Myristic acid analog YnMyr-mediated detection of NMT inhibition. Top: cells pre-incubated with or without **1**–**5** for 30 min, then pulsed with myristic acid analog YnMyr for 18 h and lysed. Bottom: NMT inhibition results in reduced of YnMyr tagging. YnMyr-tagged proteins are ligated by CuAAC to AzTB reporter, increasing the molecular weight (MW) (+1.3 kDa) and allowing fluorescent in-gel detection. (B) Effects on *N*-myristoylation revealed by YnMyr tagging in MDA-MB-231 cells. Left to right: cells exposed to DMSO (−) and **1**–**5**, in triplicate. Top to bottom: in-gel visualization of YnMyr-tagged proteins; western blot (WB) detection of NMT1, NMT2, loading control HSP90, and NMT substrate ARL1. Higher-MW form of ARL1 (dagger) indicates NMT activity. Full gels are depicted in Figure S2A. (C) Quantification of YnMyr tagging in MDA-MB-231 and HeLa cells. Tukey box-and-whiskers plot depicts relative fluorescence intensities of n = 10 bands (asterisks in B and Figure S2A). Plots based on n = 90, 90, 90, 30, 30, 30, 90, 90, 90, 30, 30, and 30 quantifications in duplicate, each individually corrected to loading control HSP90. (D) Quantification of YnMyr tagging of ARL1 in MDA-MB-231 and HeLa cells. Tukey box-and-whiskers plot depicts relative fluorescence intensity (dagger in B and Figures S2A and S3A). Plots based on n = 9, 9, 9, 3, 3, 3, 9, 9, 9, 3, 3, and 3 quantifications in duplicate, each individually corrected to loading control HSP90. (E) Sortase A-mediated detection of NMT inhibition. Top: cells exposed to DMSO or **1**–**5** for 18 h, then lysed. Bottom: NMT inhibition causes accrual of non-myristoylated N-terminal glycines, amendable for sortase A-mediated addition of a biotin-tagged ALPETG-Haa peptide, increasing the molecular weight by 0.8 kDa. (F) Effects on *N*-myristoylation revealed by sortase A-mediated peptide addition. Left to right: cells exposed to DMSO (−) and **1**–**5**, in triplicate. Top: WB detection of NMT substrate ARL1. Higher MW form of ARL1 (double dagger) indicates NMT inhibition. Full gels are depicted in Figure S2B. Bottom: loading control HSP90. (G) Quantification of sortase A-mediated peptide addition to ARL1 in MDA-MB-231 and HeLa cells. Tukey box-and-whisker plot depicts relative fluorescence intensity (double dagger in F and Figures S2B and S3B). Plots based on n = 9, 9, 9, 3, 3, 3, 9, 9, 9, 3, 3, and 3 quantifications in duplicate, each individually corrected to loading control HSP90.

We next determined the ability of **1–5** to inhibit NMT within living cells by pre-incubation in the presence or absence of DMSO, **1**, **2**, **3**, **4**, or **5** for 30 min (Figure 2A) followed by metabolic tagging with YnMyr, a myristic acid analog, for 18 h. After lysis, YnMyr-tagged proteins were ligated by copper-catalyzed azide-alkyne cycloaddition (CuAAC) to AzTB, a multifunctional reagent containing a TAMRA moiety and a biotin, allowing in-gel visualization by fluorescence (Figure 2B). Concentrations of **1–5** were chosen based published data for ca. 100% inhibition of cellular *N*-myristoylation (1 μM **1**; 0.1 μM **2**), or concentrations at which compounds at least partially reduced proliferation and metabolic activity (100 μM **3**; 30 μM **4**; and 10 μM **5**). As **1** and **2** had been previously validated using YnMyr methodology (Thinon et al., 2016, Mousnier et al., 2018), we compared **1** and **2** with **3**, **4**, and **5** individually on separate gels, in triplicate. Metabolic tagging of *N*-myristoylated proteins in MDA-MB-231 cells was potently abrogated by **1** and **2**, but not by **3–5** (Figure 2B, full gels in Figure S2A). HeLa cells responded identically (Figure S3A). Quantification of YnMyr tagging of ten different bands in both cell lines determined that **3**, **4**, and **5** provoke at most a minor reduction in YnMyr tagging (Figure 2C). Abundance of NMT1 and NMT2 was unaffected by **1–5** in MDA-MB-231 or HeLa cells (Figures 2B and S3A, respectively).

The effect of **1–5** on *N*-myristoylation of the known NMT substrate ARL1 was investigated by western blotting (Wright et al., 2010, Goya Grocin et al., 2018). The molecular weight (MW) of ARL1 (20 kDa) increases by 1.3 kDa when YnMyr is ligated by CuAAC to AzTB, resulting in a migratory shift on SDS-PAGE (Figure 2B, bottom). As expected, in cells exposed to **1** and **2** this higher MW form was absent due to inhibited NMT. Inversely, failure to inhibit cellular NMT by **3–5** was identified through the persistence of the higher MW form of ARL1, with identical observations in HeLa cells (Figures 2D and S3B). These results were orthogonally confirmed using a recently reported approach to detect in-cell NMT target engagement without the requirement for metabolic tagging (Figure 2E and Goya Grocin et al., 2018). Cells were exposed to the previous concentrations of **1–5** for 18 h, and following lysis a biotinylated labeling reagent (Biotin-ALPET-Haa peptide) was ligated by sortase A to the N-terminal glycine of proteins, including non-myristoylated NMT substrates formed as a result of inhibition. Addition of this peptide results in an MW increase of 0.8 kDa; the presence of this higher MW form of ARL1 occurred when *N*-myristoylation was inhibited by **1** and **2**, while **3**, **4**, and **5** had no effect in MDA-MB-231 cells (Figure 2F) or in HeLa cells (Figure 2G, blots in Figure S3C). It should be noted that neither YnMyr tagging nor sortase A labeling result in full conversion of ARL1 to the labeled form; this does not indicate incomplete *N*-myristoylation or incomplete inhibition but rather is due to substoichiometric incorporation of YnMyr and incomplete sortase A reactivity, as previously reported (Thinon et al., 2014, Goya Grocin et al., 2018).

Next, we expanded sortase A analysis to a broad panel of nine cell lines in which previous studies have reported using **3**, **4**, or **5** as putative NMT inhibitors (Figure S2D). In line with the aforementioned above, **3** did not inhibit NMT in Jurkat, HEK293, and NCI-H23 cells (Nadler et al., 1993, Vaandrager et al., 1996, Liang et al., 2015), **4** had no effect on MEF, PC-3, and NIH3T3 cells (Kim et al., 2017, Li et al., 2018), and **5** was cytotoxic in the absence NMT inhibition in A375, NCI-H929, and PANC1 cells (Bhandarkar et al., 2008, de la Puente et al., 2016, Díaz et al., 2016). However, both **1** and **2** engaged NMT in all cell lines tested (Figure S2D).

### Effects on the Global *N*-Myristoylated Proteome in Living Cells

**Figure 3:**
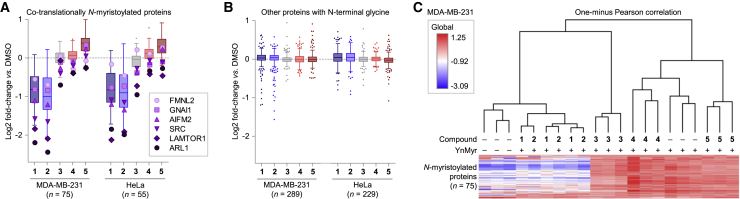
Effects on the *N*-myristoylated Proteome in Living Cells Identified by Chemical Proteomics (A) Log_2_ fold changes of co-translationally *N*-myristoylated proteins. Cells were processed as in Figure 2A, with YnMyr tagging as a measure of in-cell NMT engagement. Tukey box-and-whisker plots based on n = 75 and 55 (MDA-MB-231 and HeLa, respectively) NMT substrate identifications, in triplicate. Responses of six known co-translationally *N*-myristoylated proteins are shown for each condition. For underlying volcano plots, see Figure S4A. (B) Log_2_ fold changes of proteins with an N-terminal glycine, excluding NMT substrates, in cells exposed to **1**–**5** for 18 h. Tukey box-and-whisker plots based on n = 289 and 229 (MDA-MB-231 and HeLa, respectively) identifications in triplicates. For underlying volcano plots, see Figure S4B. (C) Hierarchical one-minus Pearson correlation clustering of co-translationally *N*-myristoylated proteins quantified in MDA-MB-231 (n = 75).

We next used quantitative chemical proteomics to identify changes in NMT substrates provoked by compounds **1**–**5**. HeLa and MDA-MB-231 cells were exposed (in triplicate) to **1**–**5**, in a setup identical to that used for in-gel analyses (Figure 2A), with the exception that YnMyr-tagged proteins were ligated to AzRB. Labeled proteins were enriched on NeutrAvidin-agarose and digested on-resin with trypsin; peptides were labeled with tandem mass tag, and analyzed and quantified by nano-liquid chromatography-tandem mass spectrometry on a Thermo Q-Exactive instrument. Inspection of volcano plots comparing fold change and significance of *N*-myristoylated proteins identified in MDA-MB-231 (n = 75, Figure S4A) and HeLa (n = 55, Figure S4B) reveals that **1** and **2** significantly inhibited *N*-myristoylation of the majority of NMT substrate proteins identified, in sharp contrast to **3**, **4**, and **5**, which had no impact on *N*-myristoylation (Figure 3A), confirming the in-gel data (Figure 2C) quantitatively at the whole-proteome level. Co-translationally *N*-myristoylated proteins showed variable rates of depletion during NMT inhibition by **1** and **2**. A myriad of factors contributes to NMT substrate sensitivities, including the affinity of the substrate protein and NMT, the *de novo* synthesis rate, rate of initiator methionine removal by methionine aminopeptidases, subcellular localization, and complex interactions at the ribosome and with other proteins (Thinon et al., 2014). Proteins with an N-terminal glycine that are not NMT substrates did not change on average compared with the control, suggesting that none of the compounds induced major proteomic changes during the 18 h of exposure (Figure 3B). Comparison of the different conditions by hierarchical one-minus Pearson correlation clustering revealed that the enrichment of *N*-myristoylated proteins in MDA-MB-231 cells exposed to **1** or **2** is very similar to that of control cells not treated with YnMyr (Figure 3C). In contrast, the response of enrichment to **3**–**5** clusters with positive control cells, i.e., YnMyr labeling in the presence of no inhibitor, providing unbiased whole-proteome evidence that **3**, **4**, and **5** do not affect NMT activity in living cells (Figure 3C). Similar hierarchical clustering of the chemical proteomics data obtained from HeLa suggests that these conclusions are common between cell lines (Figure S4C).

### Effect on Protein Synthesis, Cell Cycle, and Apoptosis

**Figure 4:**
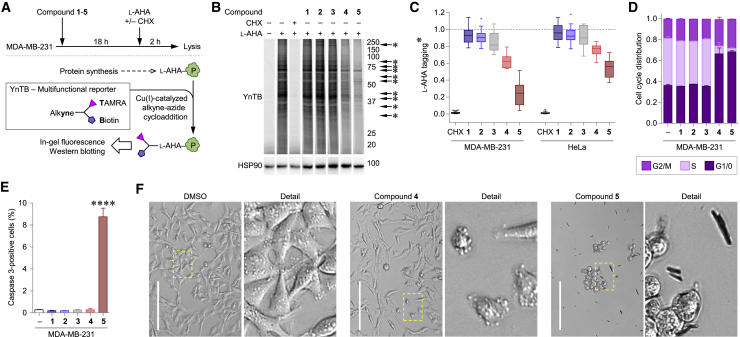
Effect on Protein Synthesis, Cell Cycle, and Cell Death in MDA-MB-231 Cells (A) Metabolic tagging of *de novo* protein synthesis with l-azidohomoalanine (l-AHA). Top: cells pre-incubated with DMSO or **1**–**5** for 18 h, then pulsed with methionine analog l-AHA for 2 h and lysed. As a positive control for protein synthesis inhibition, cycloheximide (CHX) was applied during the l-AHA pulse. Bottom: l-AHA-containing proteins are ligated by CuAAC to YnTB reporter. Protein synthesis inhibition results in reduced l-AHA incorporation and YnTB reporter fluorescence. (B) Effects on *de novo* protein synthesis revealed by l-AHA incorporation. Left to right: cells exposed to DMSO (−) and **1**–**5**. Top: in-gel visualization of l-AHA-tagged proteins. Bottom: loading control HSP90. Full gels are depicted in Figure S2C. (C) Quantification of l-AHA-tagging in MDA-MB-231 and HeLa cells. Tukey box-and-whisker plot depicts relative fluorescence intensities of n = 10 bands (asterisks in B and Figures S2C and S3C). Plots based on n = 90, 90, 90, 30, 30, 30, 90, 90, 90, 30, 30, and 30 quantifications in duplicate, each individually corrected to loading control HSP90. (D) Cell-cycle distribution of MDA-MB-231 cells after exposure to **1**–**5** for 18 h. Cells were analyzed for G_2_/M, S, and G_1_/0 through DNA content and proliferation by FACS. For the gating strategy and quantifications, see Figures S5A and S5B, respectively. Mean of n = 3 experiments ± SEM. (E) Effect on apoptosis. In MDA-MB-231 cells of (D), active caspase-3 protein staining was detected by FACS. For the gating strategy and quantifications, see Figures S5A and S5B, respectively. Mean of n = 3 experiments ± SEM. One-way ANOVA: ****p < 0.0001. (F) Bright-field micrographs depicting MDA-MB-231 cells exposed to DMSO (left), **4** (middle), and **5** (right) for 18 h. Yellow square depicts the location of the detailed area. Scale bars, 200 μm.

A reduction in co-translational *N*-myristoylation may be observed due to a failure in protein synthesis, and it is thus important to cross-validate the impact of inhibitors on protein synthesis. Cells were exposed for 18 h to **1**–**5** (1 μM **1**; 0.1 μM **2**; 100 μM **3**; 30 μM **4**; and 10 μM **5**), then washed and incubated for 2 h with the methionine analog l-azidohomoalanine (l-AHA), in the absence or presence of cycloheximide (CHX), an inhibitor of protein synthesis (Figure 4A). After lysis, l-AHA-containing proteins were ligated to YnTB and fluorescently labeled proteins resolved by gel electrophoresis (Figure 4B). Protein synthesis was not significantly reduced after 18 h with **1**, **2**, and **3**, whereas **4** and **5** provoked a significant 40%–80% decrease in l-AHA labeling, both in MDA-MB-231 and HeLa cells (Figure 4C). We next analyzed the effect of **1–5** on the cell cycle, as reduced protein synthesis may severely hamper this process. Both MDA-MB-231 and HeLa were exposed to **1**–**5** for 18 h, followed by fluorescence-activated cell sorting (FACS) analysis of DNA content and proliferation to determine the phase of the cell cycle in each cell in the tested population. As shown in Figure 4D, **4** and **5** caused a dramatic loss of cells in S phase, while increasing the populations in G_0/1/2_ and M phases, indicating that cells stop proliferating upon exposure to **4** or **5**, while **1**, **2**, and **3** had no impact on the cell cycle at 18 h. The prevalence of apoptosis was assessed next, using in the same cell populations stained for active caspase-3, the executioner protease involved in apoptosis. While **1**–**4** did not induce apoptosis in MDA-MB-231 cells, **5** increased apoptosis more than 30-fold compared with control (Figure 4E). HeLa cells, which are less sensitive to **1**–**5** compared with MDA-MB-231, showed correspondingly lower depletion of S phase and less induction of apoptosis by **4** and **5** (Figures S3D and S3E). Finally, these findings were verified by bright-field microscopy, revealing that cells of both cell lines exposed to **4** contained more rounded apoptotic cells, often with overt apoptotic blebbing (Figures 4F and S3F, respectively). Similarly, the majority of MDA-MB-231 and HeLa cells incubated with **5** exhibited this phenotype, particularly in proximity to black crystalline deposits.

## Discussion

Here we have evaluated the suitability as chemical probes of five compounds widely applied to inhibit *N*-myristoylation within living cells. *N*-Myristoylation inhibition by IMP-366 **1**, IMP-1088 **2**, 2-hydroxymyristic acid **3**, D-NMAPPD **4**, and Tris-DBA palladium **5** was identified by using a combination of quantitative whole-proteome profiling, recombinant inhibition assays, cytotoxicity, protein synthesis, cell cycling, and apoptosis. Our data show only **1** and **2** are suitable chemical probes for human NMTs, considering their capacity to inhibit recombinant NMTs and within living cells, in the absence of cytotoxicity and precipitation.

2-Hydroxymyristic acid **3** is generally applied at concentrations of 100 μM to 1 mM, whereupon a reduction of *N*-myristoylation has been described (e.g., Vaandrager et al., 1996, Tan et al., 2016, Corbic Ramljak et al., 2018). It has been proposed that **3** is activated after cellular uptake by acyl-CoA synthases to form 2-hydroxymyristoyl-CoA, a purportedly more potent inhibitor of NMTs (Paige et al., 1990, Harper et al., 1993). In our hands, however, addition of **3** at 1 mM, directly or in a mixture with BSA, resulted in precipitation in the medium and cell death (data not shown). Moreover, millimolar lipid concentrations are likely to cause off-target toxicity, as reported for porcine alveolar macrophages (1 mM, Du et al., 2010). While soluble, 100 μM **3** did not inhibit recombinant NMT1 or inhibit cellular *N*-myristoylation by a wide range of assays, and thus our data invalidate **3** as an NMT inhibitor. It seems plausible that phenotypes previously induced by **3** operate through effects on lipid metabolism, rather than by inhibition of NMT.

The application of D-NMAPPD **4** as NMT inhibitor warrants further discussion. First developed as an inhibitor for lysosomal acid ceramidase (Bielawska et al., 1996) to induce ceramide-provoked apoptosis as therapeutic strategy for melanoma (Raisova et al., 2002), **4** was recently claimed to influence c-Src activation through inhibition of NMT, at up to 30 μM **4** (Kim et al., 2017). We were unable to reproduce the stated NMT IC_50_ of 78 μM (Kim et al., 2017), and exposure of cells to 30 μM **4** did not result in changes in overall *N*-myristoylation nor a reduction of *N*-myristoylation in c-Src as measured with chemical proteomics. However, this concentration of **4** caused marked cytotoxicity within 24 h by a mechanism unrelated to NMT inhibition, illustrated by loss of protein synthesis, caspase-3 activation, and loss of metabolic activity. The order-of-magnitude difference between onset of cytotoxicity and the extrapolated IC_50_ of **4** toward recombinant NMT1 (>300 μM) further demonstrates that NMT is not a cellular target of **4**. The data are consistent with **4** potently targeting lysosomal acid ceramidase, as originally reported in various cancer cell lines at >5 μM **4**, increasing ceramide concentrations up to 12-fold and thereby inducing ceramide-driven apoptosis (Raisova et al., 2002). Our data further invalidate **4** as an NMT inhibitor, including direct evidence for failed target engagement in a selection of cell lines previously reported using **4** as a putative NMT inhibitor.

Tris-DBA palladium **5** has been previously applied as an NMT inhibitor both in living cells and *in vivo* in tumors (Bhandarkar et al., 2008, Díaz et al., 2016). It has also been claimed that **5** is more selective to NMT1 than NMT2 (0.5 and 1.3 μM, respectively; Rampoldi et al., 2012). In our analysis, **5** inhibited recombinant rNMT1 with an IC_50_ of 4.2 μM, a concentration that coincided with **5** precipitating into crystals, suggesting that **5** obstructs NMT through precipitation and not via specific interactions. Chemical proteomics further revealed that in cells exposed to 10 μM **5**, a concentration earlier reported as conditions whereby NMT was inhibited in A375 cells (Bhandarkar et al., 2008), did not prominently affect *N*-myristoylation. Indeed, the reduction of *N*-myristoylated proteins identified by chemical proteomics coincided with a marked loss of overall protein synthesis, cytotoxicity, and a 30-fold increase in apoptosis, none of which are consistent with NMT inhibition at the same time point. We noted precipitation of **5** in the growth media of multiple cell lines, most notably at concentrations over 1 μM where **5** has been suggested to cause NMT-associated cell death (Bhandarkar et al., 2008). Intriguingly, adherent cells, which would come into proximity with crystals of **5**, stopped proliferating and lost metabolic activity within 24 h, while cells in suspension were affected neither by a 10-fold higher concentration of **5** nor by the presence of crystals. These data strongly suggest that the cytotoxic effects of **5** are provoked non-specifically through proximity to crystalline deposits. Recently, **5** was incorporated into nanoparticles to circumvent the poor solubility (Elsey et al., 2019), However, the fundamental inability of **5** to inhibit cellular *N*-myristoylation while inducing marked cytotoxicity and apoptosis invalidates this compound as an NMT inhibitor.

IMP-366 **1** and IMP-1088 **2** represent chemically distinct and well-validated NMT inhibitors with defined binding modes supported by several X-ray co-crystal structures for human NMT1 and NMT2 (Thinon et al., 2014, Mousnier et al., 2018). This contrasts with **3**, **4**, and **5**, for which no X-ray co-crystal data are available. For both **1** and **2**, complete inhibition of in-cell *N*-myristoylation occurred with concentrations approximately 30- to 100-fold above the IC_50_ toward recombinant NMT1, correlating with efficacy and phenotypes observed in previous work (Mousnier et al., 2018). It should be noted that while **1** and **2** are bona fide *N*-myristoyltransferase inhibitors, our data do not comprehensively exclude additional NMTs are the only cellular targets. To identify the exact target engagement of **1** and **2**, inactive controls lacking key interactions with the NMTs should be profiled. However, structure-activity relationship data obtained during the development of **1** and **2** indicate clear on-target effects on NMT (Brand et al., 2012, Brand et al., 2014, Mousnier et al., 2018, Bell et al., 2017). Regardless, complete inhibition of in-cell *N*-myristoylation within 18 h by **1** and **2** occurred in the absence of cytotoxicity, allowing future investigations on the role and regulation of *N*-myristoylation in living cells. As noted previously, over shorter time frames pre-existing *N*-myristoylated proteins maintain cell viability even in the presence of complete NMT inhibition, while NMT inhibition induces a time-dependent reduction in proliferation and metabolic activity in cancer cell lines with prolonged exposure at 48 or 72 h.

## Significance

**The critical importance of well-characterized, on-target, cell-active chemical probes is widely appreciated across both chemical and cell biology, while invalid probes inevitably lead to misleading or invalid biological conclusions. In this work we present an in-depth evaluation of the specificity of five compounds widely applied as human *N*-myristoyltransferase (NMT) inhibitors, using a combination of enzymatic assays, quantitative whole-proteome profiling of *N*-myristoylation, cytotoxicity, in-cell protein synthesis, and cell-cycle assays. The inability of 2-hydroxymyristic acid, D-NMAPPD, and Tris-DBA palladium to inhibit cellular NMT across multiple cell types in the absence of off-target effects and cytotoxicity demonstrates the invalidity of these compounds as NMT inhibitors, and we propose that they should not be used as inhibitors of *N*-myristoylation. The results of previous studies in which these compounds are used as NMT inhibitors should be reassessed in light of these data. The evidence presented here advocates the application of IMP-366 and IMP-1088 as current best-in-class probes for human NMT, which block cellular *N*-myristoylation in the absence of off-target cytotoxicity.**

## STAR★Methods

### Key Resources Table

**Table:** 

REAGENT or RESOURCE	SOURCE	IDENTIFIER
**Antibodies**		

Rabbit anti-human ARL1	Proteintech	16012-1-AP; RRID: AB_2243131
Rabbit anti-human NMT1	Sigma-Aldrich	HPA022963; RRID: AB_1854535
Rabbit anti-human NMT2	Sigma-Aldrich	HPA001303; RRID: AB_1079494
Mouse anti-human HSP90	Santa Cruz	sc-69703; RRID: AB_2121191
Goat anti-mouse secondary antibody, HRP conjugated	Advansta	R-05071-500; RRID: AB_10718209
Goat anti-rabbit secondary antibody, HRP conjugated	Advansta	R-05072-500; RRID: AB_10719218
α-active Caspase3 antibody C92-605	BD Biosciences	550821; RRID: AB_393906

**Chemicals, Peptides, and Recombinant Proteins**		

YnMyr	Heal et al., 2011a, Heal et al., 2011b	N/A
AzTB	Heal et al., 2011a, Heal et al., 2011b	N/A
YnTB	Heal et al., 2011b	N/A
AzRB	Broncel et al., 2015	N/A
IMP-366 (compound **1**)	Frearson et al., 2010	N/A
IMP-1088 (compound **2**)	Mousnier et al., 2018	N/A
2-Hydroxymyristic acid (compound **3**)	Merck	H6771
D-NMAPPD (compound **4**)	Cambridge Bioscience	CAY10006305
Tris (dibenzylideneacetone) di-palladium (compound **5**)	Merck	328774
Human recombinant NMT1	Goncalves et al., 2012	N/A
NMT1 peptide substrate	Thinon et al., 2014	N/A
CPM	Sigma-Aldrich	C1484
Staurosporin	Sigma-Aldrich	P8833
Puromycin	Sigma-Aldrich	S4400
DAPI	Life Technologies	D1306; RRID: AB_2929482
Protease inhibitor cocktail	Roche	11873580001
Methionine-free medium	Invitrogen	A1451701
l-azidohomoalanine (l-AHA)	Sigma-Aldrich	900892
Cycloheximide	Sigma-Aldrich	239763
Recombinant Sortase A	Goya Grocin et al., 2018	N/A
Biotin-ALPETG-Haa peptide	Goya Grocin et al., 2018	N/A
TCEP	Sigma-Aldrich	C4706
TBTA	Sigma-Aldrich	678937
NeutrAvidin Agarose beads	Pierce	29201
Sequencing Grade Modified Trypsin	Promega	V5111
FxCycle Violet	ThermoFisher Scientific	F10347
Zombie NIR	Biolegend	423105
Cytofix/Cytoperm	BD Bioscience	554714
Perm/Wash Buffer	BD Bioscience	554723
DMEM, high glucose, GlutaMAX	ThermoFisher Scientific	1566016
MEM non-essential amino acids solution (100x)	ThermoFisher Scientific	11140035
HEPES (1 M)	ThermoFisher Scientific	15830080
Penicillin-Streptomycin (10000 U/mL)	ThermoFisher Scientific	15140122
Sodium pyruvate	ThermoFisher Scientific	11360070
DMEM, low glucose	Sigma-Aldrich	D6046

**Critical Commercial Assays**		

Cell Titer Blue	Promega	G8080
Protein concentration DC kit	Bio-rad	5000111
HRP Luminata Crescendo kit	Merck	WBLUR0100
TMT 10plex Isobaric Label Reagent	ThermoFisher Scientific	90111
Click-iT EdU Alexa Fluor 488 Flow Cytometry Kit	ThermoFisher Scientific	C10420

**Deposited Data**		

YnMyr proteomics dataset for HeLa and MDA-MB-231	ProteomeXchange	PXD012722

**Experimental Models: Cell Lines**		

Human cervical cancer cell line HeLa	Francis Crick Institute	ATCC CCL2; RRID: CVCL_0030
Human breast cancer cell line MDA-MB-231	Francis Crick Institute	ATCC HTB-26; RRID: CVCL_0062
Human T-cell leukemia cell line Jurkat	Francis Crick Institute	ATCC TIB-152; RRID: CVCL_0367
Human embryonic kidney cell line HEK293	Francis Crick Institute	ATCC CRL-1573; RRID: CVCL_0045
Human lung cancer cell line NCI H23	Francis Crick Institute	ATCC CRL-5800; RRID: CVCL_1547
Murine primary embryonic fibroblasts MEF	Francis Crick Institute	N/A
Human prostate cancer cell line PC-3	Francis Crick Institute	ATCC CRL-1435; RRID: CVCL_0035
Murine embryonic fibroblast cell line NIH 3T3	Francis Crick Institute	ATCC CRL-1658; RRID: CVCL_0594
Human melanoma cell line A375	Francis Crick Institute	ATCC CRL-1619; RRID: CVCL_0132
Human myeloma cell line NCI-H929	Francis Crick Institute	ATCC CRL-9068; RRID: CVCL_1600
Human pancreatic cancer cell line PANC1	Francis Crick Institute	ATCC CRL-1469; RRID: CVCL_0480

**Software and Algorithms**		

Prism 5.0	GraphPad	N/A
FlowJo	Tree star	N/A
MaxQuant 1.5.6.0	Cox et al., 2014	N/A
Perseus 1.6.2.1	Cox et al., 2014	N/A

**Other**		

Envision Xcite	PerkinElmer	N/A
PHERAstar	BMG Labtech	N/A
Acumen Cellista	TTP Labtech	N/A
Typhoon Variable Mode Imager 9500	GE Healthcare	N/A
ImageQuant LAS4000	GE Healthcare	N/A
MACSQuant VYB	Miltenyi Biotec	N/A

### Contact for Reagent and Resource Sharing

Further information and requests for resources and reagents should be directed to and will be fulfilled by the Lead Contact, Ed Tate (e.tate@imperial.ac.uk).

### Experimental Model and Subject Details

Cell lines were obtained from ATCC and verified by STR by The Francis Crick Cell Services. All cells were cultured in humidified 37°C incubators. Human cervical cancer cell line HeLa (female) and human pancreatic cancer cell line PANC1 (male) were cultured in DMEM with low glucose and 10% (v/v) FCS at a 10% (v/v) CO_2_ atmosphere. Human breast cancer cell line MDA-MB-231 (female) was cultured in DMEM with low glucose and 10% (v/v) FCS at a 5% (v/v) CO_2_ atmosphere. Human T-cell leukaemia cell line Jurkat (male) was cultured in RPMI with 10% (v/v) FCS at a 5% (v/v) CO_2_ atmosphere. Human lung cancer cell line NCI H23 (male), human myeloma cell line H929 (female), human prostate cancer cell line PC-3 (male), human melanoma cell line A375 (female), human embryonic kidney cell line HEK293 (foetal, unknown sex), murine primary embryonic fibroblasts MEF (embryonic, unknown sex) and murine embryonic fibroblast cell line NIH 3T3 (embryonic, unknown sex) were cultured in DMEM with high glucose supplemented with GlutaMAX, 10% (v/v) FCS, HEPES, sodium pyruvate, Penicillin-Streptamycin, NEAA and 2 μL/500 mL of β-mercaptoethanol, at a 5% (v/v) CO_2_ atmosphere.

### Method Details

#### Chemical Tools

The following chemical tools were synthesised as described previously: YnMyr and AzTB (Heal et al., 2011a, Heal et al., 2011b), YnTB (Heal et al., 2011b), AzRB (Broncel et al., 2015), IMP-366 **1** (Frearson et al., 2010, Thinon et al., 2014) and IMP-1088 **2** (Mousnier et al., 2018). 2-Hydroxymyristic acid **3** (Merck, H6771), D-MNAPPD **4** (Cambridge Bioscience, CAY10006305) and Tris-DBA palladium **5** (Tris (dibenzylideneacetone) di-palladium, Merck, 328774) were purchased from stated sources. All chemical tools were dissolved in DMSO, aliquoted and frozen at −20°C until further use.

#### Enzymatic HsNMT1 Assays

Full-length HsNMT1 enzyme was produced as previously described (Goncalves et al., 2012) and used at 300 ng/mL final concentration (Mousnier et al., 2018). HsNMT1 activity was determined through fluorescent detection of CoA-SH with CPM (λ_ex_ 380 nm, λ_em_ 470 nm, Envision Xcite, PerkinElmer) that formed during the *N*-myristoylation of a model peptide by HsNMT1, using myristoyl-CoA (Thinon et al., 2014). After background correction, IC_50_ values were determined by fitting the four-parametric variable slope function (GraphPad Prism 5.0), with the stated IC_50_ values being the mean of two independent assays performed in triplicate.

#### Cell Viability Assessment

In quadruplicate, HeLa (250 cells) or MDA-MB-231 (350 cells) were seeded in 384-well assay plates, and after 24 h, were incubated with 12 serial dilutions of **1-5**. After 24, 48 or 72 h of exposure, cell viability was evaluated by determination of metabolic activity and counting of cellular nuclei. Metabolic activity was assessed by addition of Cell Titer Blue (Promega), incubation at 37°C and the appropriate percentage of CO_2_ for 3 h, followed by measuring the absorbance at 490 nm using a PHERAstar (BMG LabTech, Germany). For nuclei counting, cells were fixed with 4% (w/v) paraformaldehyde at room temperature (RT) for 20 min, permeabilised with 0.2% (v/v) Triton X-100 in PBS at RT for 20 min, DNA stained with 1 μg/mL DAPI (Life Technologies, D1306) at RT for 1 h, followed by three washes with PBS. Whole well images for the total number of cells were acquired with an Acumen Cellista (TTP Labtech), using the 405 nm excitation / FL1 (420-500 nm) emission channel. The cell viability was normalised to the negative control (DMSO) and positive control (Staurosporin and puromycin), followed by fitting the four-parametric variable slope function (GraphPad Prism 5.0) to data of quadruplicate experiments.

#### Myristic Acid Analogue (YnMyr) Tagging Experiments

In triplicate, HeLa and MDA-MB-231 were seeded in 10 cm plates (10^6^ cells/plate), allowed to grow for 24 h, incubated for 30 min with DMSO, 1 μM **1**, 0.1 μM **2**, 100 μM **3**, 30 μM **4** or 10 μM **5**, followed by a metabolic tagging pulse of 20 μM YnMyr for 18 h. Then, cells were washed twice with PBS and lysed by scraping in 400 μL ice-cold lysis buffer (PBS, pH 7.4, supplemented with 1% (v/v) Triton X-100, 0.1% (w/v) SDS and EDTA-free protease inhibitor cocktail (Roche, 11873580001)). Insoluble material was separated by centrifugation (17000 *g*, 20 min, 4°C) and the supernatant was stored at −20°C until further analysis.

#### Methionine-Analogue (L-AHA) Tagging Experiments

HeLa and MDA-MB-231 were seeded in 6 cm plates (350000 or 500000 cells, respectively), allowed to grow for 18 h, and incubated with DMSO, 1 μM **1**, 0.1 μM **2**, 100 μM **3**, 30 μM **4** or 10 μM **5** for 24 h. Then, cells were washed twice with PBS and incubated for two hours in methionine-free medium (Invitrogen, A1451701), supplemented with 1 mM l-azidohomoalanine (l-AHA, Sigma-Aldrich, 900892) and with(out) 10 μg/mL cycloheximide (CHX, Sigma-Aldrich, 239763) as positive control showing abrogated protein synthesis. Cells were subsequently harvested as described before (see Myristic Acid Analogue (YnMyr) Tagging Experiments).

#### Sortase A Assay

The Sortase A assay was performed similarly as previously described (Goya Grocin et al., 2018). HeLa, and MDA-MB-231 were seeded in 10 cm plates (10^6^ cells), allowed to grow for 24 h, and incubated with DMSO, 1 μM **1**, 0.1 μM **2**, 100 μM **3**, 30 μM **4** or 10 μM **5** for 18 h. Similarly cultured, Jurkat, HEK293 and NCI-H23 cells were exposed to 100, 10 and 1 μM **3**, MEF, PC-3 and NIH 3T3 to 100, 10 and 1 μM **4** and A375, NCI-H929 and PANC-1 to 10, 1 and 0.1 μM **5**. After exposure, cells were washed twice with PBS and lysed by scraping in 400 μL ice-cold lysis buffer (50 mM Tris-HCl, pH 7.5, supplemented with 150 mM NaCl, 10 mM CaCl_2_ and EDTA-free protease inhibitor cocktail (Roche)). The homogenate was further disrupted by shearing it three times through a 30G needle. Insoluble material was separated by centrifugation (17000 *g*, 20 min, 4°C), followed determination of the protein concentration in the supernatant. Samples (75 μg total protein) were incubated with 5 μM Sortase A (Srt A) and 75 μM Biotin-ALPETG-Haa peptide for 18 h at room temperature. Hereafter the reaction was quenched with 5 mM EDTA and stored at −20°C until further analysis.

#### CuAAC Ligation and Pull-down

Samples were thawed on ice-slush and protein concentrations were determined (DC kit, Bio-rad, 5000111). Lysates were incubated with premixed CuAAC ligation reagents (100 μM AzTB (ligation to YnMyr for visualisation or with AzRB for proteomics) or YnTB (ligation to l-AHA for visualisation), 1 mM CuSO_4_, 1 mM TCEP and 100 μM TBTA) while vortexing for 1 h at RT. After quenching with 10 mM EDTA, proteins were precipitated (MeOH:CHCl_3_:H_2_O at 4:1:2), the pellet washed with cold MeOH, air-dried and resuspended in 2% (w/v) SDS, 10 mM EDTA in PBS (pH 7.4) at 10 mg/mL protein. Samples were either directly used for in-gel fluorescence detection and Westernblotting (see next section) or subjected to pull-down (see Proteomics section). Enrichment of AzTB-labelled YnMyr-labelled proteins was carried out with NeutrAvidin Agarose beads (Pierce, 29201) as described previously (Wright et al., 2014, Wright et al., 2016, Mousnier et al., 2018).

#### Gel Electrophoresis and Western Blotting

Protein samples were prepared with 5x Laemmli sample loading buffer (1 M Tris-HCl, pH 6.8, 50% (v/v) glycerol, 10% (w/v) β-mercaptoethanol, 10% (w/v) SDS, 0.01% (w/v) bromophenol blue), and boiled for 5 min at 100°C. Proteins were resolved by standard 12% (w/v) SDS-PAGE gels running at 90 V, whereafter AzTB/YnTB-labelled proteins were visualised by fluorescence-scanning on a Typhoon Variable Mode Imager 9500 (GE Healthcare) using the Cy3 (λ_ex_ 532 nm, λ_em_ 610 nm) filters to detect the TAMRA fluorophore. Hereafter, proteins were immobilised on a nitrocellulose membrane using wet blotting (Bio-RAD). Proteins were detected via Western blotting using ARL1 antibody (Proteintech, 16012-1-AP), NMT1 (Sigma Aldrich, HPA022963), NMT2 (Sigma Aldrich, HPA001303) and HSP90 (Santa Cruz, sc-69703). Using HRP-conjugated secondary antibodies (Advansta, R-05071-500 and R-05072-500) proteins were detected using the HRP Luminata kit (Merck, WBLUR0100) and chemiluminescence captured by an ImageQuant LAS4000 (GE Healthcare). Imaged protein bands were quantified using ImageJ (NIH, Bethesda) and normalized against the matching HSP90 loading control.

#### Flowcytometric Analysis

HeLa and MDA-MB-231 were seeded in 12-well plates (25000/40000 cells per well, respectively), and exposed to 0.1% (v/v) DMSO, 1 μM **1**, 0.1 μM **2**, 100 μM **3**, 30 μM **4** or 10 μM **5** for 18 h. Then, single-cell suspensions were stained with α-active Caspase3 antibody (C92-605, 550821, BD Biosciences). DNA content was stained with FxCycle Violet (ThermoFisher Scientific, F10347). Proliferation was visualised using the Click-iT EdU Alexa Fluor 488 Flow Cytometry Assay Kit (ThermoFisher Scientific, C10420) according to manufacturer’s instructions. Zombie NIR (Biolegend, 423105) was used to exclude dead cells. The cells were fixed in a 4% (w/v) solution of paraformaldehyde, permeabilised with Cytofix/Cytoperm (BD Bioscience, 554714) and washed in Perm/Wash Buffer (BD Bioscience, 554723). Samples were acquired on a MACSQuant VYB (Miltenyi Biotec) and analysed using the FlowJo software (Tree star).

#### Proteomics

Triplicate samples were prepared for MS-based proteomics of YnMyr-labelled proteins with AzRB, and analysis by nanoLC-MS/MS on a Thermo Q-Exactive instrument as described previously (Wright et al., 2014, Wright et al., 2016, Mousnier et al., 2018). After digestion with Trypsin (Promega, V5111), peptides were labelled with TMT10plex Isobaric Label Reagent (ThermoFisher Scientific, 90111). The data were processed with MaxQuant version 1.5.6.0 using the built-in Andromeda search engine (Cox et al., 2014). Peptides were identified from the MS/MS spectra by searching against the human reference with isoforms proteome database (UniProt, accessed July 2018). Cysteine carbamidomethylation was used as a fixed modification and methionine oxidation and N-terminal acetylation as variable modifications. ‘Trypsin/P’ was chosen as digestion mode enzyme, and up to two missed cleavages were allowed. The ‘match between run’ option was selected, along with ‘unique and razor peptides’ for protein quantification. TMT was set per sample group. Processed data were further analysed using Perseus version 1.6.2.1, Microsoft Office Excel 365, and GraphPad Prism 5.0. The false discovery rate was set to 0.01 for peptides and a minimum of 2 unique peptides per protein were required. The mass spectrometry proteomics data have been deposited to the ProteomeXchange Consortium via the PRIDE partner repository with the dataset identifier PXD012722.

### Quantification and Statistical Analysis

Data analysis, curve fitting and statistical testing was performed in GraphPad Prism 5.0. Recombinant human NMT1 inhibition was determined using four-parametric variable slope function on data of two experiments in triplicate. Cytotoxicity (metabolic activity and nuclei counts) was determined using four-parametric variable slope function on data of quadruple experiments. Quantification of YnMyr-tagging and l-AHA-tagging was determined by densitometry of 10 individual bands per lane per gel, for three biological replicates, and corrected to leading control HSP90. Tukey plots were generated using the quantification of a total of 90 bands for DMSO, **1** and **2**, and 30 bands for **3**, **4** and **5**. Quantification of YnMyr-tagging and Sortase-A of ARL1 occurred identically, with Tukey plots generated using the quantifications of a total of 9 bands for DMSO, **1** and **2**, and 3 bands for **3**, **4** and **5**. Analysis of proteomics data in Perseus version 1.6.2.1 was based on a false discovery rate of 0.01 and a minimum detection of 2 unique peptides per protein. Cell cycle distribution and apoptosis was determined in three experiments, with statistical significance of Caspase-3 activation determined by one-way ANOVA.

### Data and Software Availability

The mass spectrometry proteomics data have been deposited to the ProteomeXchange Consortium via the PRIDE partner repository with the dataset identifier PXD012722.

## Supplemental Information

Document S1. Figures S1–S5

Document S2. Article plus Supplemental Information
