# Supplementary material for: Validation and Invalidation of Chemical Probes for the Human N-myristoyltransferases
Source: Cell Chem Biol. 2019 Jun 20;26(6):892–900.e4. doi: 10.1016/j.chembiol.2019.03.006 (PMC6593224; doi:10.1016/j.chembiol.2019.03.006)
Supplement: Document S1. Figures S1–S5 [file mmc1.pdf]

Cell Chemical Biology, Volume *26*

Supplemental Information

Validation and Invalidation of Chemical Probes

for the Human *N*-myristoyltransferases

Wouter W. Kallemeijn, Gregor A. Lueg, Monica Faronato, Kate Hadavizadeh, Andrea Goya Grocin, Ok-Ryul Song, Michael Howell, Dinis P. Calado, and Edward W. Tate

Validation and invalidation of chemical probes for the human *N*-myristoyltransferases.

Supplementary Information

Wouter W. Kallemeijn[1,+], Gregor A. Lueg[1,2,+], Monica Faronato[1,2], Kate Hadavizadeh[1], Andrea Goya Grocin[1], Ok-Ryul Song[2], Michael Howell[2], Dinis P. Calado[2,3], Edward W. Tate[1,2]*.

[1]Department of Chemistry, Imperial College London, Molecular Research Science Hub, 80 Wood Lane, London W12 0BZ, United Kingdom.

[2]The Francis Crick Institute, 1 Midland Road, London NW1 1AT, United Kingdom.

[3]Peter Gorer Department of Immunobiology, School of Immunology & Microbial Sciences, King's College London, London SE1 9RT, United Kingdom.

[+]Authors contributed equally to this work.

*Lead contact: correspondence to e.tate@imperial.ac.uk

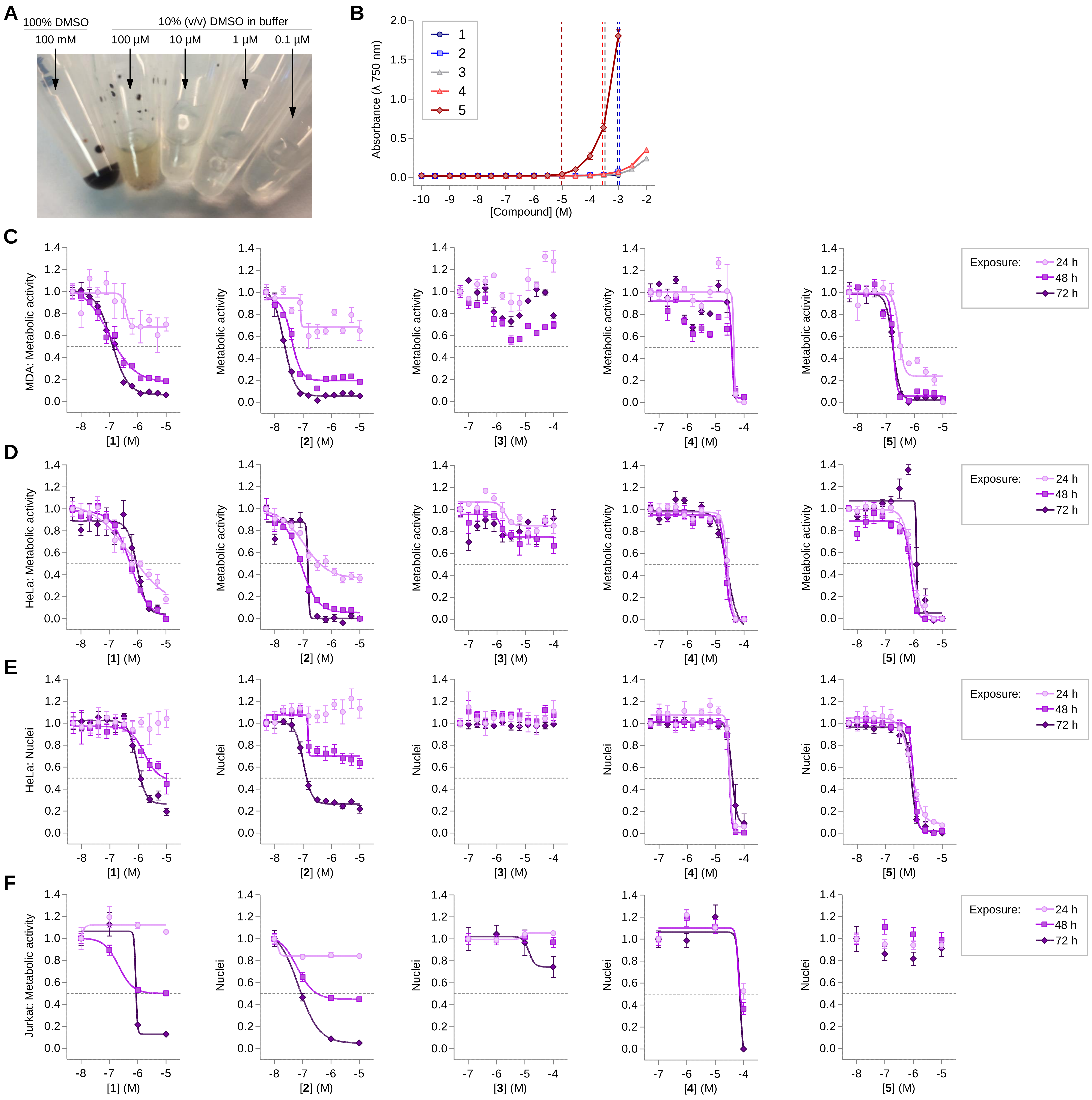
A
100% DMSO
100 mM
10% (v/v) DMSO in buffer
100 μM
10 μM
1 μM
0.1 μM
B
Absorbance (λ 750 nm)
[Compound] (M)
1
2
3
4
5
C
MDA: Metabolic activity
Metabolic activity
[1] (M)
[2] (M)
[3] (M)
[4] (M)
[5] (M)
Exposure:
24 h
48 h
72 h
D
HeLa: Metabolic activity
Metabolic activity
E
HeLa: Nuclei
Nuclei
F
Jurkat: Metabolic activity
Nuclei

Supplemental Figure 1. Effect of compounds 1-5 on recombinant protein and living cells.

Related to Figure 1.

(A) Image showing precipitation of **5** at various concentrations, within 5 min after dilution.

(B) Precipitation prevalence of compounds **1-5** in rNMT1 buffer, containing 0.1% (v/v) DMSO. Absorbance at 750 nm was determined after 15 min at room temperature. Arrows indicate prevalence of precipitates.

(C) Metabolic activity of MDA-MB-231 cells exposed to a range of **1-5** concentrations for 24, 48 and 72 h. Mean of quadruplicate measurements, ± SEM.

(D) Metabolic activity of HeLa cells exposed to a range of **1-5** concentrations for 24, 48 and 72 h. Mean of quadruplicate measurements, ± SEM.

(E) Nuclei counts of HeLa cells after exposure to **1-5** for 24, 48 and 72 h. Mean of quadruplicate measurements, ± SEM.

(F) Metabolic activity of Jurkat cells exposed to a range of **1-5** concentrations for 24, 48 and 72 h. Mean of quadruplicate measurements, ± SEM.

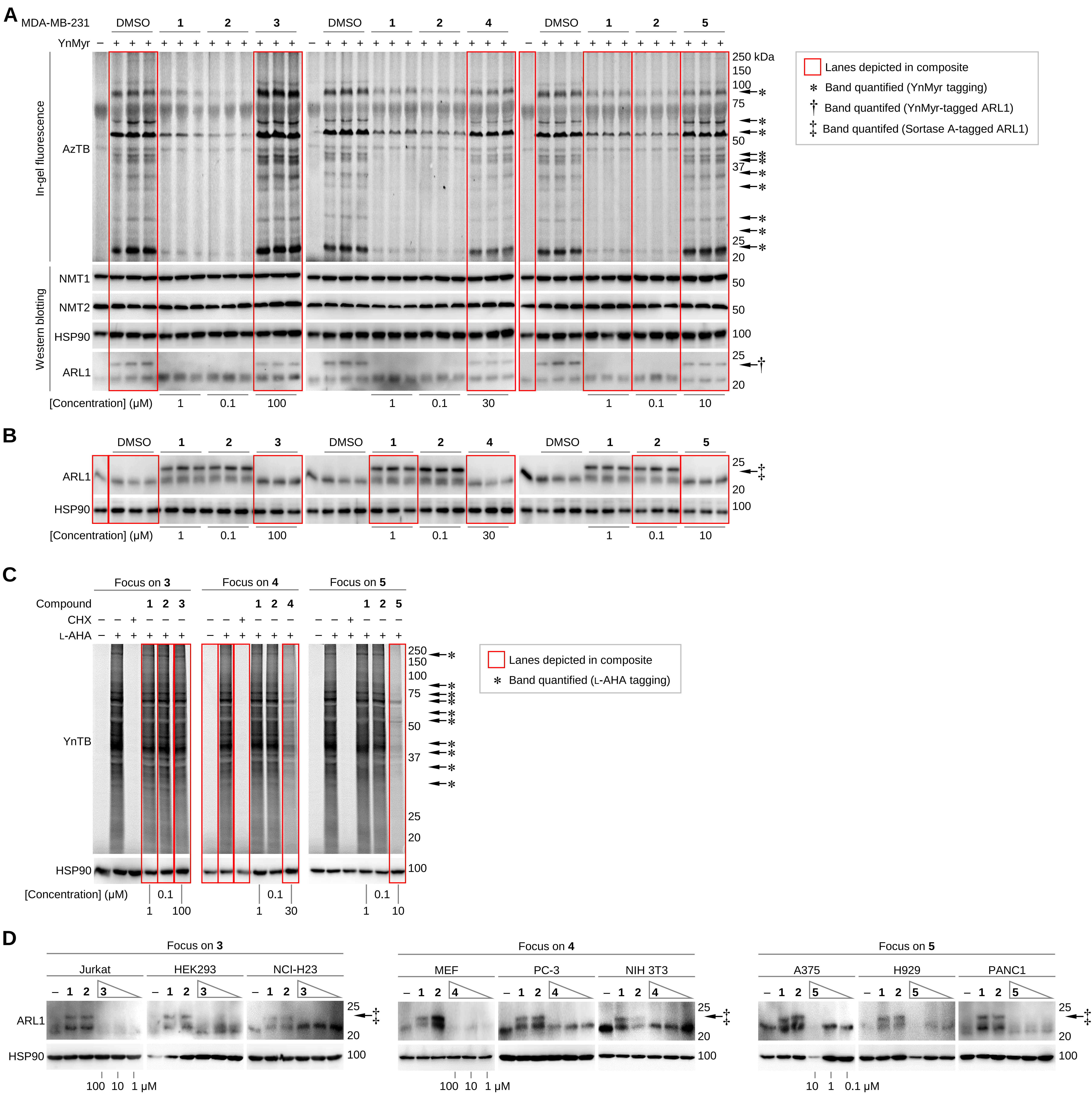
A
MDA-MB-231
DMSO
1
2
3
4
5
YnMyr
In-gel fluorescence
AzTB
Western blotting
NMT1
NMT2
HSP90
ARL1
250 kDa
150
100
75
50
37
25
20
[Concentration] (μM)
0.1
100
30
10
Lanes depicted in composite
Band quantified (YnMyr tagging)
Band quantifed (YnMyr-tagged ARL1)
Band quantifed (Sortase A-tagged ARL1)
B
C
Focus on 3
Focus on 4
Focus on 5
Compound
CHX
L-AHA
YnTB
Band quantified (L-AHA tagging)
D
Jurkat
HEK293
NCI-H23
MEF
PC-3
NIH 3T3
A375
H929
PANC1
100 10 1 μM
10 1 0.1 μM

Supplemental Figure 2. Effect of compounds 1-5 on MDA-MB-231 cells.

Related to Figures 2 and 4.

(A) Effects on *N*-myristoylation revealed by YnMyr-tagging in MDA-MB-231 cells. Left to right: cells exposed to DMSO (–), **1**, **2**, and per gel compared to **3**, **4** and **5**, in triplicate. Top to bottom: in-gel visualisation of YnMyr-tagged proteins; Westernblot (WB) detection of NMT1, NMT2, loading control HSP90 and NMT-substrate ARL1. Higher MW form of ARL1 (†) indicates NMT activity. Red squares depict regions selected for Fig 2B.

(B) Effects on *N*-myristoylation in MDA-MB-231 cells revealed by Sortase A-mediated peptide addition. Left to right: cells exposed to DMSO (–), **1**, **2**, and per gel compared to **3**, **4** and **5**, in triplicate. Top: WB detection of NMT-substrate ARL1. Higher MW form of ARL1 (‡) indicates NMT inhibition. Bottom: loading control HSP90. Red squares depict regions selected for Fig 2F.

(C) Effects on *de novo* protein synthesis in MDA-MB-231 revealed by L-AHA incorporation. Left to right: cells exposed to DMSO (–), **1**, **2**, and per gel compared to **3**, **4** and **5**, in triplicate. Top: in-gel visualisation of L-AHA-tagged proteins. Below: loading control HSP90. Red squares depict regions selected for Fig 4B.

(D) Effects on *N*-myristoylation in a panel of cell lines revealed by Sortase A-mediated peptide addition. Left to right: cells exposed to DMSO (–), **1**, **2**, and compared to different concentrations of **3** (100, 10 and 1 µM), **4** (100, 10 and 1 µM) and **5** (10, 1 and 0.1 µM). Top: WB detection of NMT-substrate ARL1. Higher MW form of ARL1 (‡) indicates NMT inhibition. Bottom: loading control HSP90.

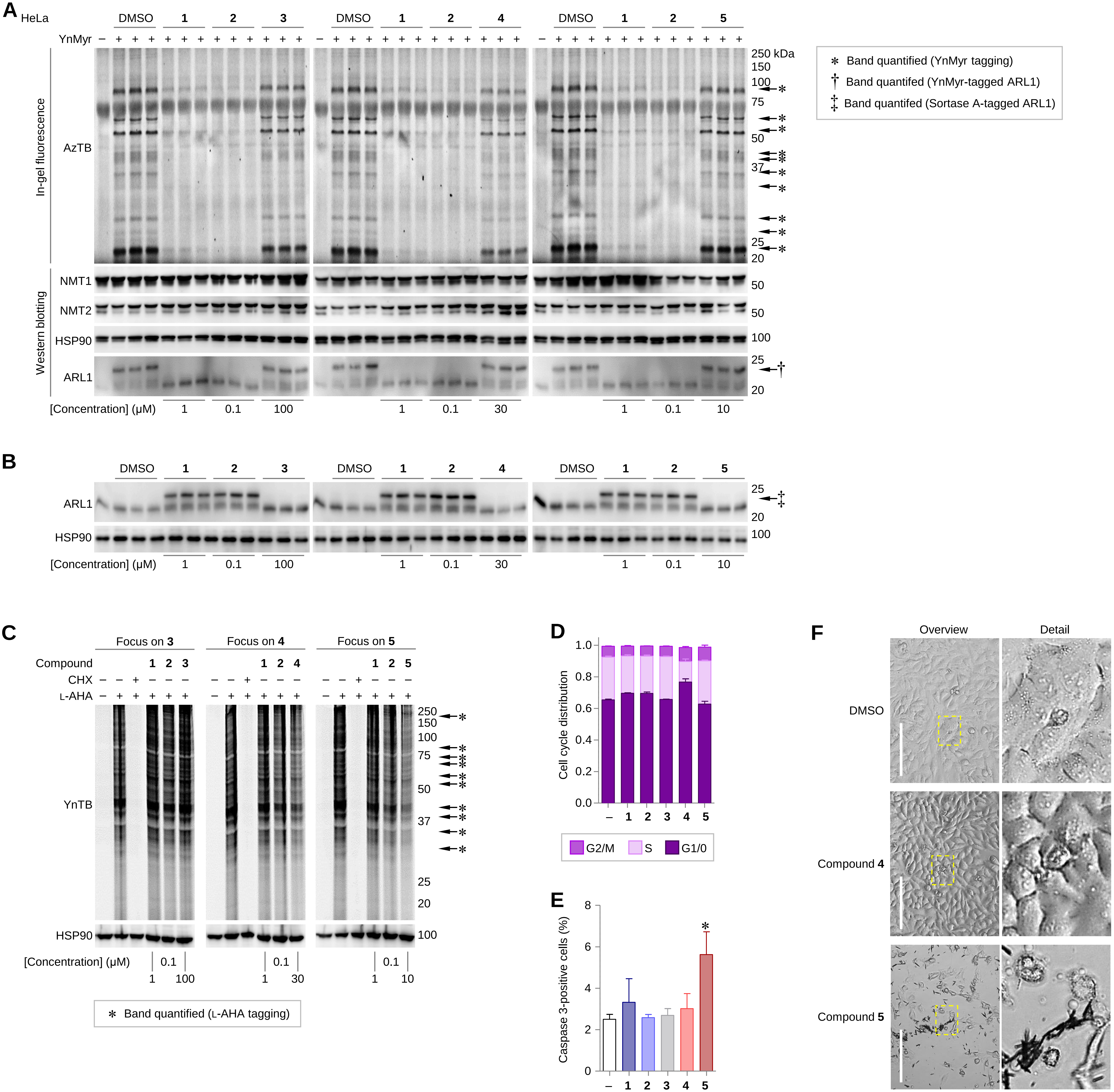
A
HeLa
DMSO
1
2
3
4
5
YnMyr
In-gel fluorescence
AzTB
Western blotting
NMT1
NMT2
HSP90
ARL1
[Concentration] (μM)
1
0.1
100
30
10
250 kDa
150
100
75
50
37
25
20
∗ Band quantified (YnMyr tagging)
† Band quantifed (YnMyr-tagged ARL1)
‡ Band quantifed (Sortase A-tagged ARL1)
B
C
Focus on 3
Focus on 4
Focus on 5
Compound
CHX
L-AHA
YnTB
∗ Band quantified (L-AHA tagging)
D
Cell cycle distribution
G2/M
S
G1/0
E
Caspase 3-positive cells (%)
F
Overview
Detail
DMSO
Compound 4
Compound 5

Supplemental Figure 3. Effect of compounds 1-5 on HeLa cells.

Related to Figures 2 and 4.

(A) Effects on *N*-myristoylation revealed by YnMyr-tagging in HeLa cells. Left to right: cells exposed to DMSO (–), **1**, **2**, and per gel compared to **3**, **4** and **5**, in triplicate. Top to bottom: in-gel visualisation of YnMyr-tagged proteins; Westernblot (WB) detection of NMT1, NMT2, loading control HSP90 and NMT-substrate ARL1. Higher MW form of ARL1 (†) indicates NMT activity.

(B) Effects on *N*-myristoylation in HeLa cells revealed by Sortase A-mediated peptide addition. Left to right: cells exposed to DMSO (–), **1**, **2**, and per gel compared to **3**, **4** and **5**, in triplicate. Top: WB detection of NMT-substrate ARL1. Higher MW form of ARL1 (‡) indicates NMT inhibition. Bottom: loading control HSP90.

(C) Effects on *de novo* protein synthesis in HeLa revealed by L-AHA incorporation. Left to right: cells exposed to DMSO (–), **1**, **2**, and per gel compared to **3**, **4** and **5**, in triplicate. Top: in-gel visualisation of L-AHA-tagged proteins. Below: loading control HSP90.

(D) Cell cycle distribution of HeLa after exposure to **1-5** for 18 h. Cells were analysed for G2/M, S and G1/0 through DNA content and proliferation by FACS. For the gating strategy and quantifications, see Fig S5C and S5D, respectively.

(E) Effect on apoptosis. In HeLa cells of (D), active Caspase-3 protein staining detected by FACS. For the gating strategy and quantifications, see Fig S5C and S5D, respectively.

(F) Brightfield micrographs depicting HeLa cells exposed to DMSO (top), **4** (middle) and **5** (bottom) for 18 h. Yellow square depicts the location of the detail area. Scale bar is 200 μm.

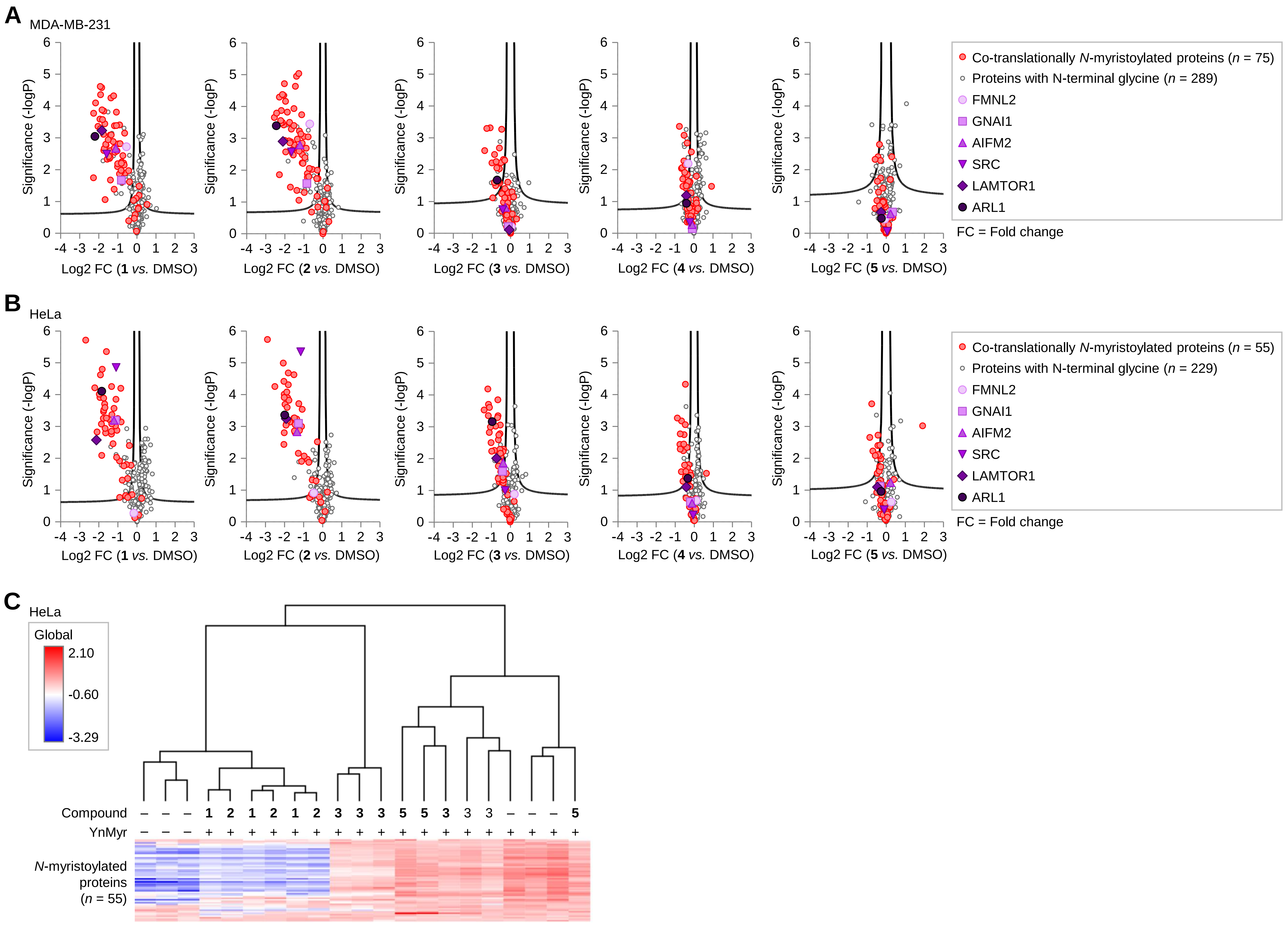
A
MDA-MB-231
Significance (-logP)
Log2 FC (1 vs. DMSO)
Log2 FC (2 vs. DMSO)
Log2 FC (3 vs. DMSO)
Log2 FC (4 vs. DMSO)
Log2 FC (5 vs. DMSO)
Co-translationally N-myristoylated proteins (n = 75)
Proteins with N-terminal glycine (n = 289)
FMNL2
GNAI1
AIFM2
SRC
LAMTOR1
ARL1
FC = Fold change
B
HeLa
Co-translationally N-myristoylated proteins (n = 55)
Proteins with N-terminal glycine (n = 229)
C
HeLa
Global
2.10
-0.60
-3.29
Compound
YnMyr
N-myristoylated proteins (n = 55)

Supplemental Figure 4. Chemical proteomics on YnMyr-tagged proteins.

Related to Figure 3.

(A) Volcano plots for MDA-MB-231 showing the Log2 fold-change and significance of proteins identified after enrichment of YnMyr-tagged proteins and chemical proteomics. Depicted are co-translationally *N*-myristoylated proteins (red, *n* = 75) and proteins with N-terminal glycines (grey, *n* = 289). Left to right: Compound **1-5** versus DMSO. Responses of six known co-translationally *N*-myristoylated proteins are shown for each condition. A negative Log2 fold-change indicates less protein identified in cells with compound compared to cells treated with DMSO only. Black lines depict the significance threshold FDR (0.01).

(B) Volcano plots for HeLa, as above. Co-translationally *N*-myristoylated proteins (red, *n* = 55) and proteins with N-terminal glycines (grey, *n* = 229).

(C) Hierarchical one-minus Pearson correlation clustering of the co-translationally *N*-myristoylated proteins quantified in HeLa (*n* = 55).

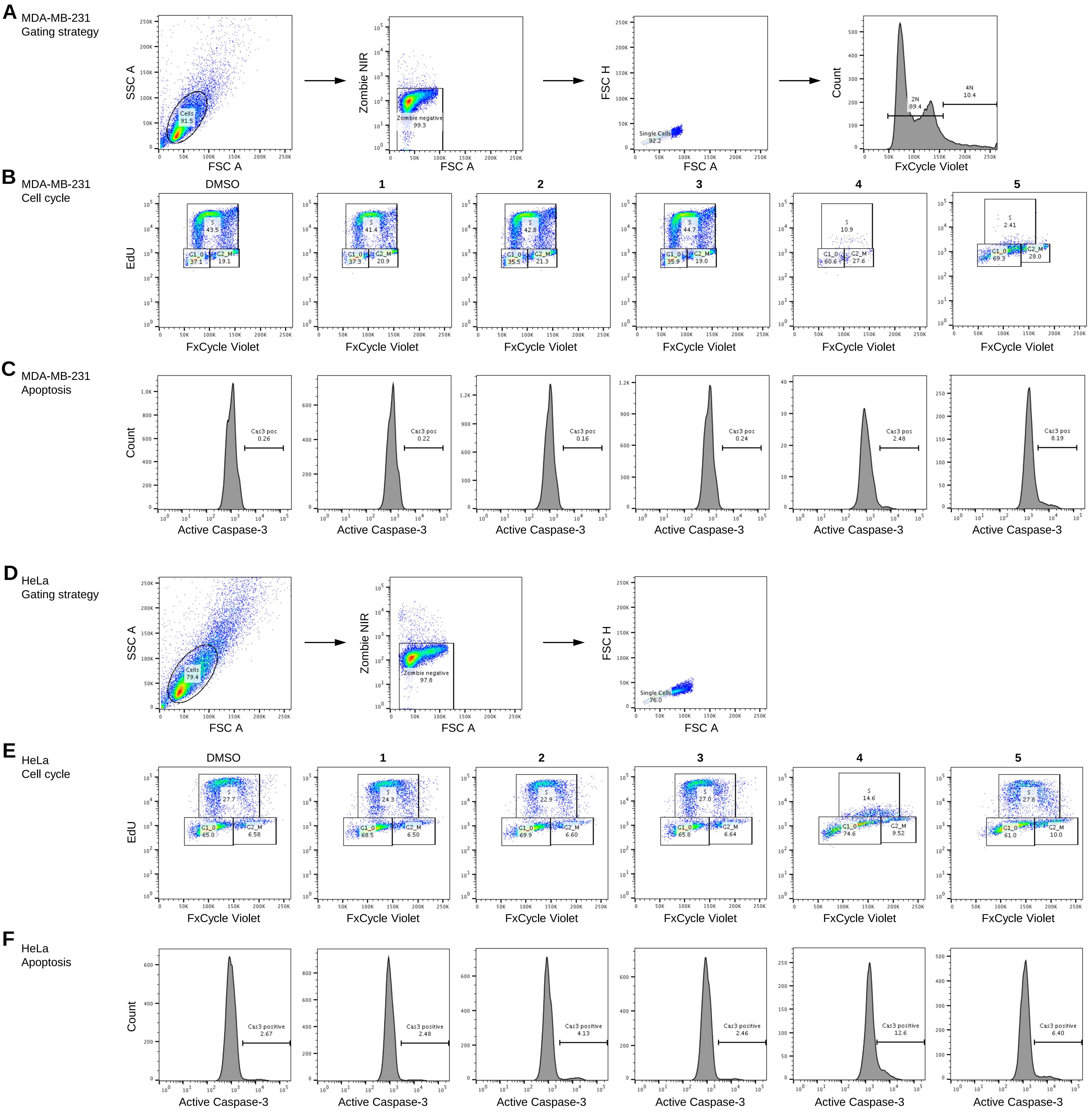
A MDA-MB-231 Gating strategy
SSC A
FSC A
Cells 91.5
Zombie NIR
Zombie negative 99.3
FSC H
Single Cells 92.2
Count
FxCycle Violet
2N 89.4
4N 10.4
B MDA-MB-231 Cell cycle
DMSO
1
2
3
4
5
EdU
S 43.5
G1_0 37.1
G2_M 19.1
S 41.4
G1_0 37.3
G2_M 20.9
S 42.8
G1_0 35.5
G2_M 21.3
S 44.7
G1_0 35.9
G2_M 19.0
S 10.9
G1_0 60.6
G2_M 27.6
S 2.41
G1_0 69.3
G2_M 28.0
C MDA-MB-231 Apoptosis
Active Caspase-3
Cas3 pos 0.26
Cas3 pos 0.22
Cas3 pos 0.16
Cas3 pos 0.24
Cas3 pos 2.48
Cas3 pos 8.19
D HeLa Gating strategy
Cells 79.4
Zombie negative 97.8
Single Cells 76.0
E HeLa Cell cycle
S 27.7
G1_0 65.0
G2_M 6.58
S 24.3
G1_0 68.5
G2_M 6.50
S 22.9
G1_0 69.9
G2_M 6.60
S 27.0
G1_0 65.8
G2_M 6.64
S 14.6
G1_0 74.6
G2_M 9.52
S 27.8
G1_0 61.0
G2_M 10.0
F HeLa Apoptosis
Cas3 positive 2.67
Cas3 positive 2.48
Cas3 positive 4.13
Cas3 positive 2.46
Cas3 positive 12.6
Cas3 positive 6.40

Supplemental Figure 5. Effects on cell cycle and apoptosis in intact living cells.

Related to Figure 4.

(A) Gating strategy for MDA-MB-231 cells. Left to right: gating of cell size, gating of living cells (Zombie NIR negative), gating of single cells and gating of the cell population with 2n only.

(B) FACS plots showing DNA content (FxCycle Violet) staining compared to proliferation (EdU incorporation) in MDA-MB-231. Left to right: effects to cells exposed to DMSO, or **1-5** for 18 h.

(C) FACS plots showing active Caspase-3 protein staining detected by FACS. Left to right: effects to cells exposed to DMSO, or **1-5** for 18 h.

(D) Gating strategy for HeLa cells. Left to right: gating of cell size, gating of living cells (Zombie NIR negative), gating of single cells and gating of the cell population with 2n only.

(E) FACS plot showing DNA content (FxCycle Violet) staining compared to proliferation (EdU incorporation) in HeLa. Left to right: effects to cells exposed to DMSO, or **1-5** for 18 h.

(F) FACS plot showing active Caspase-3 protein staining detected by FACS. Left to right: effects to cells exposed to DMSO, or **1-5** for 18 h.
